# Meaning Making as a Central Mechanism of Dignity Therapy for Older Adults With Cancer

**DOI:** 10.1093/geroni/igaa057.1352

**Published:** 2020-12-16

**Authors:** Emily Mroz, Carma Bylund-Lincoln, Rachel Wisolmerski, Diana Wilkie, George Fitchett, George Handzo, Harvey Chochinov, Susan Bluck

**Affiliations:** 1 University of Florida, Gainesville, Florida, United States; 2 Rush University Medical Center, Chicago, Illinois, United States; 3 HealthCare Chaplaincy Network, New York, New York, United States; 4 University of Manitoba, Winnipeg, Manitoba, Canada

## Abstract

Nearly 500,000 older Americans die a cancer-related death each year (National Vital Statistics Report, 2018). Following a diagnosis of a serious illness like cancer, maintaining a sense of dignity is central to a patient’s wellbeing. Dignity Therapy (DT) was recently introduced as an intervention to enhance dignity for terminally ill patients (Chochinov et al., 2005). This therapy provides patients opportunities to foster a sense of dignity though making meaning of their lives (Hack et al., 2010). To date, whether meaning-making actually occurs as a central mechanism of effective DT has not been tested. The current study investigates (i) how often and in what forms meaning-making occurs during DT, and (ii) how patients’ baseline feelings of dignity relate to meaning-making during DT. Participants were 25 male and female cancer outpatients (M age = 63.08; SD = 5.72). They completed the Patient Dignity Inventory (Chochinov et al., 2008) and then participated in Dignity Therapy with a trained provider. Sessions were audio recorded, transcribed, and reliably content-analyzed for meaning-making using an established coding scheme (Park & Folkman, 1997). Content-analysis revealed that all patients made meaning of past life events at least once (range: 1-12 occurrences). Multiple forms of meaning-making emerged, with Finding Benefit and Personal Growth most common. Patients reporting more dignity-related distress prior to DT showed greater meaning-making during the DT session (r = .46, p < 0.05). This study provides foundational evidence that meaning-making is a key mechanism of Dignity Therapy, helping older adults with cancer enhance dignity at end-of-life.

